# Anthropogenic impacts drive niche and conservation metrics of a cryptic rattlesnake on the Colorado Plateau of western North America

**DOI:** 10.1098/rsos.160047

**Published:** 2016-04-27

**Authors:** M. R. Douglas, M. A. Davis, M. Amarello, J. J. Smith, G. W. Schuett, H.-W. Herrmann, A. T. Holycross, M. E. Douglas

**Affiliations:** 1 Department of Biological Sciences, University of Arkansas, Fayetteville, AR, USA; 2 Illinois Natural History Survey, University of Illinois, Champaign, IL, USA; 3 Life Sciences, Arizona State University, Tempe, AZ, USA; 4 Biology, Georgia State University, Atlanta, GA, USA; 5 Natural Resources and Environment, University of Arizona, Tucson, AZ, USA

**Keywords:** climate change, *Crotalus cerberus*, drainage vicariance, environmental niche modelling, wildfire

## Abstract

Ecosystems transition quickly in the Anthropocene, whereas biodiversity adapts more slowly. Here we simulated a shifting woodland ecosystem on the Colorado Plateau of western North America by using as its proxy over space and time the fundamental niche of the Arizona black rattlesnake (*Crotalus cerberus*). We found an expansive (= end-of-Pleistocene) range that contracted sharply (= present), but is blocked topographically by Grand Canyon/Colorado River as it shifts predictably northwestward under moderate climate change (= 2080). Vulnerability to contemporary wildfire was quantified from available records, with forested area reduced more than 27% over 13 years. Both ‘ecosystem metrics' underscore how climate and wildfire are rapidly converting the Plateau ecosystem into novel habitat. To gauge potential effects on *C. cerberus*, we derived a series of relevant ‘conservation metrics' (i.e. genetic variability, dispersal capacity, effective population size) by sequencing 118 individuals across 846 bp of mitochondrial (mt)DNA-ATPase8/6. We identified five significantly different clades (net sequence divergence = 2.2%) isolated by drainage/topography, with low dispersal (*F*_ST_ = 0.82) and small sizes (2*N*_ef_ = 5.2). Our compiled metrics (i.e. small-populations, topographic-isolation, low-dispersal versus conserved-niche, vulnerable-ecosystem, dispersal barriers) underscore the susceptibility of this woodland specialist to a climate and wildfire tandem. We offer adaptive management scenarios that may counterbalance these metrics and avoid the extirpation of this and other highly specialized, relictual woodland clades.

## 1. Introduction

Geomorphic processes drive major ecosystem shifts, whereas more gradual changes in the natural environment promote their diversification. This synergistic ontogeny forms the baseline for a contemporary perspective on ecosystem evolution where environmental transformations are both shared and codependent with resident biodiversity [1]. It also yields a series of ‘ecosystem metrics’ that not only document the manner by which ecosystems transition over time, but also the concomitant constraints that can emerge with these shifts [2], particularly when transformations are inordinately forced.

Similarly, a series of ‘conservation metrics’ can be inferred for resident biodiversity to guide conservation efforts, such as building corridors to reconnect populations and ecosystems now fragmented by anthropogenic activities [3]. Alternatively, individuals can be translocated among isolated areas to re-establish extirpated biodiversity components or promote genetic rescue of dwindling populations [4], but such actions come with caveats [5]. Conservation metrics are most easily derived from molecular data that, in turn, can determine the origin of populations (via coalescence among clades), their levels of connectivity (by quantifying gene flow), as well as their persistence over time (by estimating genetic diversity, demographic trends and effective sizes) [5,6].

Conservation and ecosystem metrics are inherently relevant for biodiversity management, and a clear mapping of these ‘biodiversity-to-ecosystem’ linkages is especially germane for conservation in the Anthropocene [7]. Both provide a template for adaptive management, with options that can span from conserving or restoring damaged ecosystems [8], to coping with those deemed novel and thus seemingly intractable [9]. Many of these metrics are also employed to estimate climate change velocity, or the rapidity with which ecosystems are driven towards alternative equilibria. Such studies underscore the link between global refugia and areas of low velocity [10], and also predict the speed at which a particular species must migrate so as to maintain its niche [11]. This mapping also has relevance for those biodiversity elements considered short-range endemics (as herein) [12]. However, dispersal capacities and population sizes for such species are often misjudged or underestimated [5]. This, in turn, diminishes the predictive power of the mapping, particularly when vulnerabilities of ecosystem are increased and conserved niches additionally compressed. Many biodiversity elements that reside in such situations are now recognized as ‘conservation-reliant’ ([13], and references therein), necessitating the derivation of accurate conservation and ecosystem metrics so as to blunt impending impacts.

**Figure 1.**
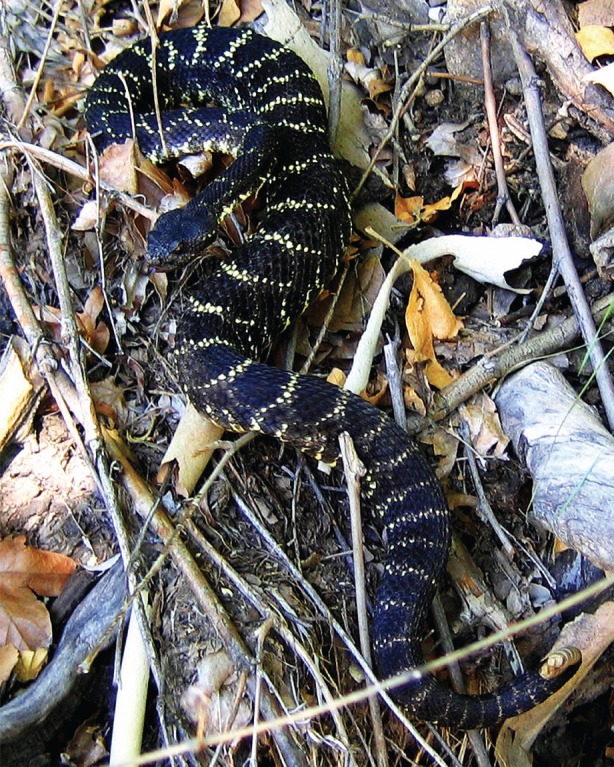
*Crotalus cerberus*, Santa Catalina Mountains, Pinal County, Arizona. Picture taken by Roger A. Repp, 6 August 2008, and published with permission.

We employed several approaches to evaluate transitioning of the forested Colorado Plateau ecosystem of western North America, and to gauge the response by resident, range-restricted biodiversity to these shifts. Our intent was to evaluate conservation and niche metrics of a relatively sedentary but charismatic species as a potential bookmark for other niche-conserved species that may experience similar landscape-level interactions and disturbance histories. We first derived an ecological niche model (ENM) for a cryptic, but social study species (the Arizona black rattlesnake, *Crotalus cerberus*) [14,15] (figure 1), and used it as a proxy to evaluate the shifting forested habitat of the Plateau over time and in response to a fluctuating climate [12].

In this context, the geographical range of *C. cerberus* is considered a spatial representation of its fundamental niche [16], and the simulation of these data in both an historic and predictive framework is interpreted as a species-to-ecosystem map. ENMs are critical both for conservation planning and resource management, and are often used to determine those species likely to adapt *in situ* versus those that have the potential to disperse to a more suitable niche, or even face extirpation [17]. ENMs can also predict if refugia, as currently designated, will continue into the future or will instead become a sink for both ecosystem services and coevolved species [18]. We further extended our biodiversity-ecosystem map by estimating the contemporary vulnerability of the wooded Plateau to severity and extent of wildfire over the past 13 years.

Additionally, we quantified the conservation metrics of *C. cerberus* (i.e. the extent of its genetic variability, its capacity for dispersal, the size of its effective populations, its potential for bottlenecks) by sequencing two rapidly evolving mitochondrial (mt)DNA genes. Based on the insights gained from estimating these niche and conservation metrics, we offer potential policy enhancements that could facilitate the management of both the ecosystem and its biodiversity moving forward.

## 2. Material and methods

### 2.1. The fundamental niche of the Arizona black rattlesnake

We used ENMs to frame the study ecosystem and predict its potential range shift going forward. In this sense, ENMs represent the fundamental niche of a species, and are recognized as stable traits within and across lineages [19]. They essentially define that set of abiotic environmental conditions within which a species can survive and maintain viable populations. This approach is particularly well suited for *C. cerberus*, whose ectothermic physiology is tightly linked to environmental factors.

From 1998 to 2005, two teams collected genetic samples of 118 *C. cerberus* from across its range [20]. Capture coordinates for these samples were supplemented with locality coordinates from all available museum specimens, and the entire dataset (*N* = 302) was imported into MaxEnt [21] and parsed among training/testing sets (*N* = 227/75, respectively). Nineteen bioclimatic variables were obtained from the WorldClim database [22] and a correlation matrix derived to identify eight biologically meaningful but uncorrelated variables [23]. These are: annual mean temperature; mean diurnal range; maximum temperature in the warmest period; minimum temperature of the coldest period; annual temperature range; mean temperature of the warmest quarter; mean temperature of the coldest quarter and annual precipitation. Three of these were also employed in a previous climate model derived for Plateau grasslands [24], thus lending credence to the extrapolation of fundamental niche to ecosystem metrics.

Because *C. cerberus* was previously recognized as a member of a species-complex [25], and given the inherent complexity of modelling intra-specific entities [26–28], we excluded from calibration those areas where subspecies potentially co-occurred. In so doing, we adjusted for the fact that conspecifics (congenerics) with similar habitat requirements may also perhaps exist therein. ENMs consistently perform better, and most often predict larger areas of suitable conditions, when the potential for sub-taxonomic structure is accommodated.

The fundamental niche for *C. cerberus* was derived from 10 147 points and averaged across 15 replicates of 5000 iterations each. We then derived a predictive post-Pleistocene species envelope, as well as one for 2080, based on a conservative climate projection of the Coupled Global Climate Model 2 (CGCM2) [29], as averaged and iterated above. BioClim variables were assessed for their relative contributions while information content was evaluated using the jackknife procedure. We also tested a suite of standard regularization multipliers (i.e. values of 1–10, 15 and 20) to ensure veracity of the projected climate envelope model [30–32]. The improvement in the fit of the model was evaluated using ENMTools [33,34], and mean distributional estimates for both models were then imported into ARCGIS v. 10 for derivation of climate envelopes and core habitat areas (per [5]).

### 2.2. Ecosystem vulnerability as a metric

Wildfire was first documented in the Silurian (420 Ma [35]), and it subsequently dominated the highly flammable savannahs within which hominins coevolved over millennia. Reciprocity has occurred of late, particularly in western North America, where anthropogenic activities shape ecosystems by promoting the magnitude and intensity of wildfire [36,37]. This situation has been augmented by extreme drought, high wind and rugged topographies [38], and also compounded by controversial fire suppression programmes [39]. The consequence? A precipitous decline in biodiversity on the Plateau as burned acreage quadruples with every degree the temperature rises [40]. Those biodiversity elements less vagile are clearly most susceptible [41].

To evaluate the vulnerability of the forested ecosystem that contains *C. cerberus*, we obtained from the Web [42] locations burned by wildfire in the 13 most recent years. From this, we were able to dissect out and evaluate the number of hectares incinerated by wildfire.

### 2.3. Derivation of conservation metrics

For genetic analyses, whole blood was collected and preserved (approx. 0.1 ml) from 118 *C. cerberus* often sampled singly (or in pairs) across an elevated, forested and topographically rugged ecosystem [25]. These logistic difficulties, coupled with the natural history of the Crotalinae (per [43]), prevented an accumulation of samples sufficient for broad population genetic analysis and, in turn, restricted our choice of molecular markers to mtDNA. Previously derived protocols [44] guided the sequence analysis of the mtDNA ATPase8/6 genes, the construction of a minimum spanning network of haplotypes, and the derivation of net sequence divergences (sd) among clades. A Bayesian phylogenetic analysis (BA) [45] consisted of two runs of five chains sampled every 1000 generations, and terminated with average standard deviation among split frequencies less than 0.001. Parameters/trees were estimated from 10 million generations (less than 30% burn-in) and visualized as a majority-rule consensus tree, with prairie rattlesnake (*C. viridis*) as outgroup [25].

We applied five separate runs in a coalescent-based Markov Chain Monte Carlo (MCMC) approach [46] to estimate clade-specific values for *Θ* (= 2*N*_e_ × *m*) and *M* (= mutation-scaled immigration rate = *m*/*μ*) using 5 million generations/eight chains, four adaptively heated, with 10 000 burn-in samples discarded. Five *Θ*-values were averaged per clade, multiplied by each (of four) pairwise immigration values then derived as clade-specific mean effective population size (female 2*N*_ef_). Analysis of molecular variance (AMOVA) and *F*_ST_ values were computed among clades [47] or groups, as defined by landscape features (rivers and basins), with *p-*values derived from 1000 permutations.

## 3. Results

### 3.1. Ecosystem and conservation metrics

**Figure 2.**
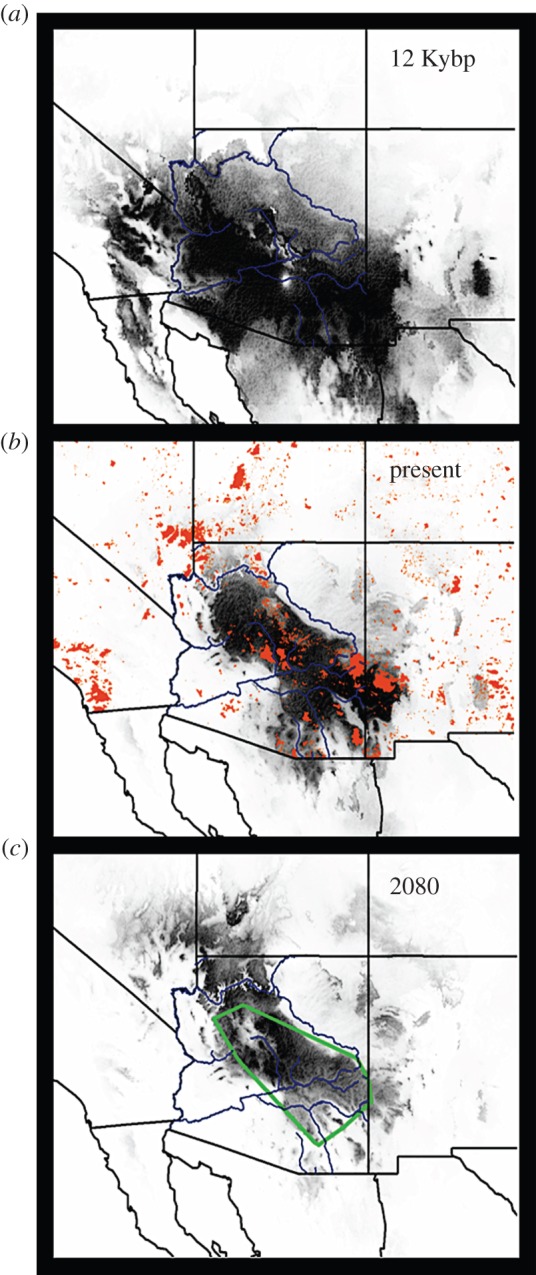
Three environmental niche models depicting core habitat for 302 *Crotalus cerberus* within the forested ecosystem of the Colorado Plateau, southwestern North America: (*a*) = 12 Kya (*b*) = present (orange = 13 most recent years of wildfire locations/ha = 27% of range) and (*c*) = 2080 (green polygon = current distribution).

The modelled shifting of the *C. cerberus* distribution showed congruent trends over time and within the forested ecosystem of the Colorado Plateau, with an historic configuration (figure 2*a*) condensing sharply into the present (figure 2*b*). With less stringent climate predictors [21], the ENM further condensed to higher elevations at the northern periphery of the core area, but with a major extension to the extreme northwest (2080: figure 2*c*; contemporary range of *C. cerberus* framed in green). Of particular note is that the shifting core area for this species is truncated topographically by an impenetrable Grand Canyon and Colorado River, effectively eliminating any potential for range expansion concomitant with a shifting niche distribution.

**Figure 3.**
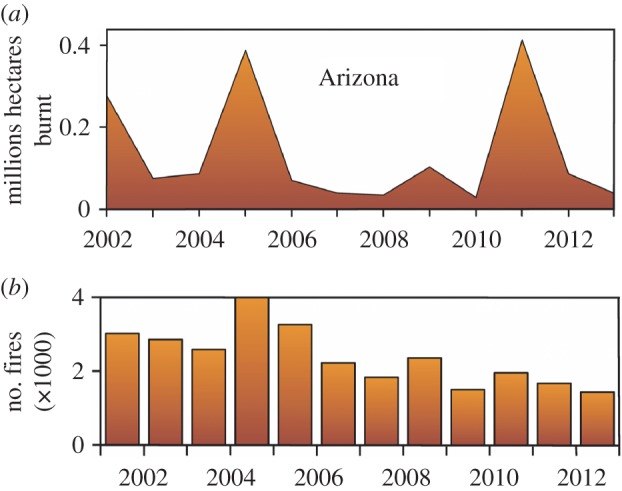
(*a*) Frequency plot depicting millions of hectares burned in Arizona by year (13 most recent); (*b*) histogram presenting the number of fires (×1000) over the same temporal span. http://landfire.cr.usgs.gov/viewer/.

Ecosystem vulnerability was estimated as the frequency and intensity of wildfire that has occurred on the Colorado Plateau of Arizona over the last 13 years (figure 3). These data were then topographically depicted in orange as burned hectares in figure 2*b*. The duration and extent of wildfire has reduced the forested niche of *C. cerberus* (and concurrently its ecosystem) by greater than 27%.

**Figure 4.**
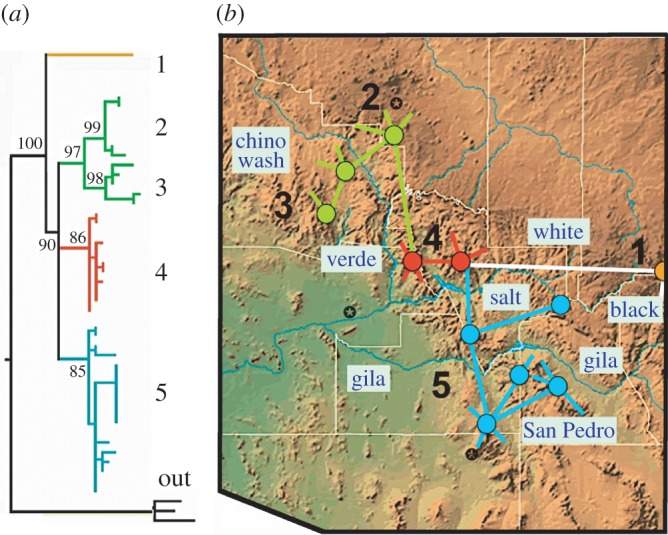
(*a*) Bayesian phylogenetic analysis of *Crotalus cerberus* (Colorado Plateau/southwestern North America) depicting 48 haplotypes/five clades derived from 846 bp of mtDNA-ATP8/6. Outgroup = *Crotalus viridis*; (*b*) haplotype network derived from the same data as above but projected onto Arizona topography, with rivers (open boxes) demarcating clades. AZ map produced by Map Resources (http://www.mapresources.com/).

The conservation metrics of *C. cerberus* were estimated from the evaluation of mtDNA sequence data, and a Bayesian Analysis recovered five distinct clades at greater than 85% (figure 4*a*). A haplotype network projected onto a topographic map (figure 4*b*) revealed rivers as vicariant barriers. These were: Black River (Clade-1, NM); Salt and Gila rivers (Clade-5, southeastern AZ); Verde River (Clade-4, central AZ); East and West Clear creeks (Clade-2, northern AZ) and Big Chino Wash/Agua Fria (Clade-3, northwestern AZ). All clades diverged significantly with regard to pairwise sequence divergence and *F*_ST_ values (table 1).

**Table 1.** Pairwise %-sequence divergences (top triangle) and *F*_ST_-values (lower triangle) among five clades of *Crotalus cerberu*s (Colorado Plateau, southwestern North America), as derived from 846 bp of mtDNA-ATP8/6 (*N* = 118 individuals). All values differ significantly (*p* < 0.005).

clade	5-Blue	4-Red	3-Green	2-Green	1-NM
5-Blue	X	1.0	2.4	2.7	2.2
4-Red	0.65	X	2.0	2.3	2.1
3-Green	0.83	0.83	X	1.5	2.7
2-Green	0.84	0.83	0.81	X	2.9
1-NM	0.80	0.80	0.90	0.88	X

Two AMOVAs significantly partitioned genetic diversity (*p* < 0.0001) by (i) clade (81.7%) and (ii) topography as demarcated by rivers (69.6%), but not according to drainage basins (40.9%), again underscoring the presence of rivers as physical barriers to gene flow. Conversely, average 2*N*_ef_ values were depressed despite considerable historic migration, and ranged from 1.5 to 8.3, indicating that clade divergence was significantly driven by small size and increased isolation (table 2).

**Table 2.** Average genetic diversity parameters for five clades of *Crotalus cerberus* (Colorado Plateau, southwestern North America) derived from 846 bp of mtDNA-ATP8/6 sequenced (*N* = 118 individuals). Values: theta = (av. Th); migration = (av. *M*); female effective population size = (av. 2*N*_ef_); net-sequence divergence = (av.%sd); *F*_ST _= (av. *F*_ST_).

clade	av. Th	av. *M*	av. 2*N*_ef_	av.%sd	av. *F*_ST_
5-Blue	0.0184	79.2	1.5	2.1	0.78
4-Red	0.0289	287.3	8.3	1.8	0.78
3-Green	0.0139	323.7	4.5	2.1	0.84
2-Green	0.0153	367.4	5.6	2.3	0.85
1-NM	0.0144	435.6	6.3	2.4	0.85
average	0.0182	298.6	5.2	2.2	0.82

## 4. Discussion

Historic data are increasingly employed to comprehend long-term ecological change and to provide a context within which biodiversity conservation can be framed. As such, historic data are necessary to estimate how ecosystems transition over time, and to evaluate the corresponding response by resident biodiversity [48]. They also have a role in guiding ecosystem restoration, particularly for establishment of habitat corridors that promote connectivity and sustain long-term responses in an ever-shifting Anthropocene [1].

Our goal in this study was to understand the influence of climate and landscape on a niche-conserved, range-restricted species and apply it as a species-ecosystem proxy to gauge potential impacts on other species with similar ecologies. We first delineated the fundamental niche of *C. cerberus* as distributed at end-of-Pleistocene, then projected these data into the present, as well as predicting a conservative trajectory as configured over the next 50 years. The unique properties of molecular genetic data then allowed us to examine how tractable the dispersal of *C. cerberus* was within this ecosystem, and to reconstruct a geographical context for diversification. As a matrix within which to juxtapose the phylogeographic distribution across the Plateau, a snapshot of its deep history was first needed.

### 4.1. The matrix of deep history

An historic perspective on landscape diversification has importance in that it can help gauge more contemporary impacts, as well as offer a prognosis for its trajectory in a changing climate [49]. The dynamic landscape of arid southwestern North America has been shaped by tectonism and climatic oscillations, but is now subjected to steadily increasing anthropogenic pressures [5,6,50]. Key geomorphic events that occurred on the Plateau during the Late Miocene–Early Pliocene were the collapse of the southwest Basin and Range physiographic province and the integration of the Colorado River. These synergistic occurrences were concomitant with the ongoing uplift of the Plateau, which in turn provoked deep incisions within its antecedent (i.e. previously formed) Plateau streams. For example, a well-defined structural trough guided the uppermost Gila River (figure 4) into a closed, ephemeral basin before the river integrated westward to the Colorado River [51]. Other Late Miocene drainages (i.e. Salt River, Verde River; figure 4) similarly flowed via deeply incised canyons into closed basins that soon spilled into headwater-eroding canyons during a more pluvial Late Pliocene [52]. These events were pivotal with regard to biodiversity evolution in that they promoted landscape diversification (figure 2*a*) and accentuated a vicariant separation of clades (figure 4*a*).

This pattern occurred repeatedly in southwestern North America [25,44,48], such that taxa with broader distributions were fragmented over time by the synergistic effects of climate and tectonism, whereas more geographically restricted taxa were relatively unaffected [48,53]. Thus, given the contemporary distribution reflected by our study species (green polygon, figure 2*c*), we expected and tested for a shallow genetic structure by quantifying molecular diversity within regions having a common phylogenetic or biogeographic history. Rather than a shallow diversification, we found instead a topographically embedded haplotype network of five significantly isolated clades, each with reduced gene flow, as underscored by migration rates, *F*_ST_ values, and female effective population sizes (tables 1 and 2). In addition, these diversification patterns aligned quite closely with drainage evolution (figure 4*b*).

Once we understood the strong influence of hydrographic and tectonic processes on the phylogeographic patterns of regional biotas [54], we could then evaluate the vulnerabilities of this ecosystem with regard to more contemporary impacts, and how the latter may, in turn, provoke a turnover of woodland habitat on the Plateau. We were also interested in gauging the magnitude and extent of these potential impacts with regard to *C. cerberus*.

### 4.2. Wildfire as an ecosystem regulator

Fire has become a global management tool, and it is often used in this context to regulate ecosystems [55]. Planned burns can provide benchmarks from which to infer impacts produced by spontaneous wildfire. The patterns that emerge, while intuitively appealing, are often ill defined [56–59], but with an occasional emergent property. Small mammals, for example, are a key component of forested food webs, and as such are prey items for *C. cerberus*. Their abundance within Sierra Nevada (CA) forests was greater within unburned plots [60], largely due to the presence of over-story and a shelter-providing ground layer, a phenomenon that translates broadly across vertebrate groups [61]. Furthermore, small mammals became extirpated when fire occurred more than once at a single location over a 5-year span [60]. Clearly, wildfire has serious and substantial effects on the persistence of favourable habitat, and on those biodiversity elements that form the prey of apex predators such as *C. cerberus*.

As a management tool, fire moderates the environment [55], but with the caveat that it must be controlled so as to avoid local extinctions. In this sense, high-intensity fires are quite lethal for small vertebrates, whether as management endeavours [62] or naturally occurring (per figure 3*a*). Wooded canyons and steep slopes burn more intensely due to an elevated fuel accumulation, and this reverberates post-fire in that surviving individuals subsequently remain in subterranean retreats for protracted periods [63]. The occurrence of wildfire and its intensity are covariates that not only impact the vegetation and prey base, but also the subsequent behaviours of prey and predators.

### 4.3. A juxtaposition of metrics

Conservation planning and fire management can be juxtaposed, in that both have readily achievable goals that are linked to decision-making tools and operational guidelines. However, each requires sustained data so as to identify critical questions, and to specify appropriate means of adjudication [64,65]. An optimal fire history is one such example that can be modelled for a given area by developing a biodiversity index so as to define species-specific responses [66]. Conservation objectives can emerge as a direct product, but with the caveat that life history, demography, and a fine-grained distribution are *a priori* requirements (per [5]). An unfortunate downside to this approach is that such data are often lacking for many relictual species, to include *C. cerberus*.

From an historic context, wildfire in southwestern North America was a ‘rejuvenator’ of mountainous ecosystems [66], but its effects post-settlement were deemed deleterious and it was vigorously suppressed as a consequence [39]. It has again rebounded as a significant disturbance [67] with an expansive future as an ecosystem ‘converter’ [68] in synergy with climate change [69]. Communities are now driven towards new equilibria that contain novel species-compositions that are resilient to a relapse [70].

## 5. Conclusion

In this study, we examined genetic structure of a niche-conserved species so as to understand the manner by which climate and landscape have influenced its past, contemporary and predicted distributions. In doing so, we found reduced gene flow, limited dispersal and significant vicariance as the ecosystem shifted in elevation rather than latitude. Furthermore, our data demonstrated discrete, significantly different clades whose genetic diversities cannot withstand the erosive effects of wildfire [71], particularly when the capacity to disperse is limited not only by life history but also by landscape barriers.

The goal of management should be to maintain levels of gene flow and efforts in this regard can be guided by conservation metrics, as inferred from molecular data. In this sense, the network of genetic connections as derived among clades is the web that sustains their continued evolution (figure 4). However, limitations are imposed by the ecosystem. For example, the strong synergy between severe drought and wildfire [72] is an unfortunate harbinger for eventual extinction of clades, or even species-extirpation on the southern Plateau, as ponderosa pine/pinyon-juniper woodlands are converted into novel habitat [70].

An apt example of how climate change and wildfire can impact a cryptic, niche-conserved and short-range rattlesnake is provided by *Crotalus willardi obscurus* in the sky-islands of southwestern North America [5]. Long-term recapture data, combined with demographic and niche modelling, demonstrated a survival probability that is significantly impacted by wildfire, and furthermore, an extinction vortex driven by small population demographics. Both aspects translate well to *C. cerberus* and its larger geographical range. Both taxa are embedded within the rugged topography of a forested ecosystem that inhibits gene flow, constrains effective population sizes, and induced significant clade diversification. The two species are strikingly complementary with regard to ecosystems and life histories, and scant extrapolation is required to suggest that *C. cerberus* will have an extinction trajectory concomitant with that of *C. w. obscurus* in the near time.

In the context of adaptive management, how can the unique biodiversity of the Colorado Plateau ecosystem be appropriately conserved? Three opportunities present themselves: first, the conservation status of *C. cerberus* should be designated as ‘threatened’ under the Endangered Species Act (ESA), so as to more appropriately leverage ecosystem management for the Plateau. This would allow the U.S. Fish & Wildlife Service (FWS) to develop regulatory protections adjusted to the needs of the species, rather than as a protective blanket afforded to those designated as ‘endangered.’ In this sense, limited (but not complete) protection is provided under the ESA, and this in turn promotes additional (and entrepreneurial) conservation options. As part of this process, ‘critical habitat’ could be designated so as to effectively promote recovery goals [73].

These considerations promote a second opportunity. The Plateau should be promoted as a prime example of ecosystem vulnerability, as driven by climate change and its accompanying wildfire component [74]. The mapping of *C. cerberus* within its ecosystem underscores the fact that consequences are apparent for both when uncertain climatic shifts are manifest. Finally, other unique biodiversity elements on the Plateau should also be recognized so as to promote not only public awareness but also perceptions of stakeholders regarding ecosystem vulnerabilities. These actions may offer the Plateau and its biodiversity a brief respite, but hopefully enough time to allow substantive ecosystem-level initiatives in the context of region-specific mandates [44,49,50].

## Data accessibility

GenBank accession number: KX095994-KX096036.
